# FAMILY DECISION-MAKING IN SERIOUS ILLNESS SCENARIOS: ACCURACY AND PERCEIVED ACCURACY OF ADULT CHILD SURROGATES

**DOI:** 10.1093/geroni/igac059.1388

**Published:** 2022-12-20

**Authors:** Jessica Hahne, Brian Carpenter

**Affiliations:** Washington University in St. Louis, St. Louis, Missouri, United States; Washington University in St. Louis, St. Louis, Missouri, United States

## Abstract

Adult children often help older adults make important decisions in serious illness situations, but previous research suggests adult children are not uniformly accurate in predicting parents' preferences. In this project we examined the accuracy with which adult children (N= 68; M age= 45; 69.1% female) were able to predict medical preferences of their parents (N= 34; M age= 75; 94.1% female). Parents expressed their preferences in four serious illness scenarios, and children predicted parents' preferences. Parents and children also estimated the accuracy of children's predictions and the frequency of communication about medical care. On average across all scenarios, children predicted parents' preferences accurately 42.3% of the time. Parents were willing to take more risk with treatment for more severe disease, as their children predicted, although children generally overestimated parents' willingness to live with increasing disability (M difference = 9.77%). Overall, children were poor at predicting their own accuracy (Rs = -.017, p= .893). Parents were slightly more insightful about their children's accuracy (Rs= .261, p=.034), but there was no association between accuracy and parent or child estimate of frequency of conversations about medical preferences (Parent Rs= .067, p=.586 ; Child Rs= .164, p= .182). These findings suggest that neither are adult children highly accurate at predicting parents' medical preferences, nor do they or their parents have a precise sense of their accuracy. Results from this study may inform interventions to help families communicate about advance care planning.

